# Quality assurance using quality indicators for prevention and early detection of cervical cancer in certified gynaecological dysplasia units and consultancies

**DOI:** 10.1007/s00404-024-07694-w

**Published:** 2024-08-29

**Authors:** Lena Steinkasserer, Simone Wesselmann, Jens Quaas, Matthias W. Beckmann, Christian Dannecker, Jens Hachenberg, Matthias Jentschke, Peter Hillemanns

**Affiliations:** 1 Department of Gynaecology and Obstetrics, Hannover Medical School, Carl-Neuberg-Straße 1, 30625 Hannover, Germany; 2 German Cancer Society e.V., Berlin, Germany; 3 Working Group Cervical Pathology & Colposcopy, Hanseatic City, Stralsund, Germany; 4 Department of Gynecology and Obstetrics, Erlangen University Hospital, Erlangen, Germany; 5 Department of Obstetrics and Gynecology, University Hospital, Augsburg, Germany

**Keywords:** Cervical cancer, Screening, Prevention, Quality indicators, Quality assurance, Dysplasia unit, Dysplasia consultancy

## Abstract

**Purpose:**

Cervical cancer is the fourth most common cancer in women worldwide. A successful screening concept for cervical cancer reduces the incidence and mortality of cervical cancer. Quality indicators (QIs) derived from the screening guidelines for cervical cancer and used by the certified dysplasia units and dysplasia consultancies are evaluated in this paper. The aim of this paper is to present the current data from the annual reports of these units and consultancies.

**Methods:**

The results of the basic data and indicators for the audit year 2022 in the gynaecological dysplasia consultancies and units are presented. In 2022, 84 dysplasia consultancies and 42 units were audited. 40 units and 84 consultancies are included in the annual report. QI outcomes for patients treated in certified dysplasia units and dysplasia consultancies are analysed. Median, overall proportion, and standard deviation were calculated for each QI.

**Results:**

The indicator year 2021 was analysed, which was audited in 2022 and evaluated in 2023. A total of nine QIs were analysed. Most target goals were met by the 84 certified dysplasia consultancies and by the 40 dysplasia units. The QIs evaluated are implemented to a very high degree. The targets for the three QIs were achieved by both the dysplasia consultancies and the units in at least 95% of the certified centres (QI 1: 100%, QI 2: 95%, QI 3: 100%; QI 1: 100%, QI 2: 97%, QI 3: 100%, respectively). The presentation of patients to the tumour board by the consultancies/units is working; the units are attending the tumour board more regularly than in previous years. Where the target was not met, the auditors issued deviations or reduced the duration of the certificate. The cases are discussed intensively in the sense of an individual case analysis and with the determination of measures on-site.

**Conclusions:**

The targets for the various indicators were largely met by the dysplasia consultancies and units in the 2022 audit year. The certification of gynaecological dysplasia consultancies/units which have to cooperate with certified gynaecological cancer centres, has for the first time ensured the continuity of healthcare from prevention and early diagnosis to treatment of gynaecological cancers.

## What does this study add to the clinical work

**Table:** 

With the certification of gynaecological dysplasia consultancies /units, the continuum of healthcare from prevention to treatment of gynaecological cancers in certified gynaecological cancer centres was ensured for the first time. The monitoring of defined quality indicators is intended to ensure that the quality of care in the dysplasia consultancies and units is guaranteed. QIs can be used to establish guideline-compliant treatment and motivate practitioners to review their treatment outcomes. The results of the QI reports are reported to the medical guideline development groups and provide information on whether and how the recommendations made are implemented in everyday clinical practice. They thus provide a stimulus for the further development of the corresponding guideline.	

## Introduction

Cervical cancer is the fourth most common cancer in women worldwide [1]. In 2020, approximately 4.640 women in Germany developed cervical cancer [2]. In 1971 a national screening program for cervical cancer by cytological examination was introduced. Since then, the incidence rate of cervical cancer has declined sharply over decades [3, 4]. Established screening has kept the incidence and mortality of cervical cancer low for the past 15 years [5, 6]. However, organized screening is needed to further reduce the incidence and mortality of cervical cancer. In 2018, the Federal Joint Committee (Gemeinsamer Bundesausschuss—G-BA) was mandated to establish an organized national screening program in Germany, which includes an organized invitation procedure and an evaluation of the screening program. The pre-existing, non-organized annual screening by cytological smear continues for women aged 20–34 years. For women over 34 years of age, a combined cytological smear and HPV test is offered every three years [7].

In Germany, the German Guideline Programme in Oncology (GGPO) develops evidence- and consensus-based screening and treatment guidelines are developed for all tumour entities. In this program, the derivation of quality indicators from strong recommendations of the guideline is a mandatory component. These Quality Indicators (QIs) are used in the certified consultancies, units and centres of the German Cancer Society. The interaction between the development of guidelines and quality indicators and their use and evaluation in the certified care structures is described as the Oncology Quality Cycle and is a result of the National Cancer Plan in Germany.

In Germany, there is an "Oncology Quality Cycle" for quality assurance and effective implementation of guideline recommendations in everyday oncological screening, diagnosis and care, including the use of derived QIs. These are derived from the recommendations of the S3 guidelines of the Oncology German Guideline Program [8]. The quality assurance and improvement process for certified cancer centres, units and consultancies arise from compliance with guideline recommendations, which is captured by the implementation rate of QIs. Compliance with these and other guideline recommendations is monitored and evaluated by the certification system of the German Cancer Society [9].

The German Cancer Aid (Deutsche Krebshilfe–DKG) has developed and introduced a system for the certification of oncology centres, organ cancer centres and screening consultancies/units. The requirements for certification are developed and continuously refined in certification commissions. The Certification Commission for Gynaecological Cancers consists of experts in gynaecological oncology who define the requirements and quality indicators for certification based on evidence-based guideline. These QIs are used together with the guideline QIs (see above) in the certification process [10].

Similar certification systems also exist in the rest of Europe and in the United States of America.

In Europe, the European Federation for Colposcopy (EFC) has a Quality and Standards Group that oversees the development and updating of quality indicators and other aspects of quality assurance for colposcopy in Europe. The main task of this group is to review the relevance and feasibility of current quality indicators. This will be achieved in collaboration with the national colposcopy societies and their representatives. In addition, other aspects of quality within a colposcopy service will be identified. Where appropriate, this will be done in collaboration with other organizations such as IFCPC, ESGO, EBCOG, ECCA and the European Commission [11]. Although there is no national certification program for colposcopy in the United States, the American Society for Colposcopy and Cervical Pathology (ASCCP) strongly recommends completion of the Colposcopy Mentorship Program (CMP) as a minimum requirement for training a colposcopist. The CMP is divided into three levels, all of which must be completed in order to be certified [12].

The main goal of the certification system is to continuously improve the prevention, early detection, diagnosis and treatment of cervical cancer. The certification system is being implemented for all tumor entities. This article presents and analyses the results of cervical dysplasia units and consultancies. The QI results of the gynaecological cancer centres for patients with cervical cancer are described by Stuebs et al. [13].

At the end of 2022, there were 42 certified gynecologic dysplasia units and 285 certified gynecologic dysplasia consultancies. 40 of the 42 units are included in the annual report of the dysplasia units. As dysplasia consultations are only required to be re-certify every three years, only 84 consultations were audited in 2022.

## Methods

### Included certified dysplasia units and consultancies

Prerequisite for participation in a certification process is the cooperation of the respective dysplasia unit and consultancy with a DKG-certified Gynaecological Cancer Centre.

There are differences between dysplasia units and dysplasia consultancies. This is not least due to the number of documented colposcopies and histologies to be examined. While dysplasia units must have a minimum number of excisions, dysplasia consultancies are free to perform excisions.

The certification system for dysplasia consultancy is person-based, whereas the certification system for dysplasia units is person- and facility-based. Dysplasia consultancies only have to provide evidence for three of the nine QIs. Dysplasia units, however, must provide fulfilment of all nine QIs. From 2024, dysplasia consultancies must also state QI 4–9 if they carry out excisions.

### Certification requirements and audit process

An overview of the certification program for gynaecological dysplasia consultancies and units was presented by Quaas et al. [13].

Certification is based on a catalogue of requirements, which summarizes the minimum quantitative and qualitative requirements that must be met to be certified. In addition, a datasheet contains QIs that are either derived from the S3 guideline or defined by the certification commission for gynaecological cancers. The minimum requirements for each designated physician in the dysplasia unit are at least 100 cases with abnormal colposcopic findings and at least 50 cases with histological confirmation. For the entire unit, the minimum requirements are at least 300 cases with abnormal colposcopic findings, at least 150 cases with histological confirmation and at least 100 excisions. The minimum requirements for the dysplasia consultancy are at least 100 cases with abnormal colposcopic findings and at least 30 cases with histological confirmation. No excisions are required. Dysplasia units are audited annually on-site, while the dysplasia consultancies are audited every three years in a paper-based rather than on-site certification process. OnkoZert, the independent certification institute of the DKG organizes the auditing process on behalf of the DKG. The audits are carried out by trained gynaecological-oncological medical doctors.

### Data collection

For certification each dysplasia unit and dysplasia consultancy must, among other criteria, document compliance with the QI. The results of the QI must be reported annually by the dysplasia units and every three years by the dysplasia consultancies to the independent certification institute OnkoZert. The data sheets of the individual facilities are analysed and checked for plausibility. Target values or plausibility limits are defined for the individual indicators. Certified units/consultancies must provide a justification if their results deviate from these target values limits.

Auditors review the reported data and justifications and inspect patient records to verify the data. The verified data are published in the benchmarking reports.

The data presented here are based on patient data from 2021. These were reviewed in the audits in 2022 and are summarized in the 2023 annual report.

### Data analyses

A descriptive analysis of case distribution, patient numbers, and indicator definitions was performed. QI outcomes for patients treated in 2021 were analysed. For each QI, the median and the total proportion of units/consultancies achieving the target value of the QI were calculated. Data were presented in tables. The results, nominator, denominator, units/consultancies achieved and target value were presented here.

Statistics were performed using Data WhiteBox. This data analysis tool was specially developed by OnkoZert. A p-value ≤ 0.05 is considered significant. No independent evaluation of the QI for dysplasia consultancies was performed for the treatment years 2018 and 2019, so these analyses are missing.

## Results

At the end of 2022, there were 42 certified gynaecological dysplasia units and 285 certified gynaecological dysplasia consultancies. 40 of the 42 facilities are included in the annual report of the dysplasia units. Four dysplasia units were certified for the first time in the 2022 audit year (including one certificate with a reduced validity period); 17 units were successfully recertified (Two certificates with a reduced validity period). In 2022, 84 dysplasia consultancies were audited (person-related, reduced requirements, document evaluation/case review every 3 years). All nine QIs were included in the datasheet for dysplasia units and consultancies in the treatment year 2021. However, dysplasia consultancies only have to fulfil the first three QIs, whereas dysplasia units have to fulfil all nine QIs. The following nine QIs were included in the datasheet for the certified units/consultancies "Presentation at tumour board" [QI 1], "Participation in interdisciplinary tumour board/centre event" [QI 2], "Documentation (at least one sketch) of the visibility of the squamous cylindrical epithelial border" [QI 3], "Performance of expert colposcopy" [QI 4] and "Abnormal findings of excision" [Q 5], " Details pathology report" [QI 6], "Proportion of R0 resection for CIN III" [QI 7], "Follow-up care after excision" [Q 8] and "Proportion of cold knife conization of excisions" [QI 9]. No independent evaluation of the QI for dysplasia consultancies was prepared for the treatment years 2018 and 2019, so these analyses are missing.

The Definition of nominator and denominator are shown in Table 1.

**Table 1:** Definition of numerator and denominator of QIs

QIs	
1. Presentation at tumour board	Numerator: no. of patients presented at the tumour conference Denominator: no. of patients with an invasive cancer
2. Participation in interdisciplinary tumour board/centre event	Participation in tumour board of the gynaecological cancer centre
3. Documentation (at least one sketch) of the visibility of the squamous cylindrical epithelial border	Numerator: no. of patients in which the squamous epithelial-cytoplasmic border was documented (at least sketch) Denominator: no. of patients with colposcopy of the cervix uteri
4. Performance of expert colposcopy	Numerator: no of. patients for whom a expert colposcopy was performed preoperatively in the dysplasia unit Denominator: no. of patients who have undergone an excision of the cervix uteri
5. Abnormal findings of excision	Numerator: no. of patients with leading histology ≥ CIN 2 Denominator: Patients who have undergone an excision of the cervix uteri
6. Details pathology report	Numerator: no. of patients with complete, written histologic assessment of the excision (= type and size for all lesions (not metric), vertical and horizontal extension for invasive lesions, resection margins, distance of lesion to endocervical resection margin in mm) Denominator: Patients who have undergone excision of the cervix uteri, vulva, vagina
7. Proportion of R0 resection for CIN III	Numerator: no. of patients with R0 resection Denominator: no. of patient with excision and histologic findings CIN III
8. Follow-up care after excision	Numerator: no. of patients with recommendation for follow-up once 6–12 months after excision Denominator: no. of patients who have undergone an excision of the cervix uteri
9. Proportion of cold knife conization of excisions	Numerator: no. of patients with knife conization Denominator: Patients who have undergone excision of the cervix uteri

### Annual report of certified gynaecologic dysplasia consultancies [14]

In 2022, 84 dysplasia consultancies were audited. The results for the dysplasia consultancies are shown in Table 2.

**Table 2:** Quality indicators for dysplasia consultancies (treatment years 2017, 2020, 2021)

Quality indicator		2021				2020				2017			
		Results/ cases	Median nominator	Median denominator	Consultancies meeting TV	Results/ cases	Median nominator	Median denominator	Centres meeting (TV)	Results/ cases	Median nominator	Median denominator	Centres meeting (TV)
1	Tumour board presentation (TV: > = 90%)	453/ 456	4 [1–39]	4 [1–39]	97,33% (73/75)	517/ 525	4 [1–88]	4 [1–92]	98,57% (69/70)	514/ 522	6 [0–100]	6 [0–100]	95,45% (42/44)
2	Participation in interdisciplinary tumour board/ cancer centre event (TV: > = 4 participants/year)	986	5 [2–105]		95,24% (80/84)	1016	8 [4–52]		100,00% (74/74)	555	5 [0–47]		95,74% (45/47)
3	Documentation (at least one sketch) of the visibility of the squamous cylindrical epithelial border (TV: > = 85%)	20,782/ 21,275	189 [72–1077]	195,5 [72–1077]	100,00% (84/84)	13,614/ 13,940	126 [45–950]	129 [49–950]	100,00% (74/74)	9036/ 9464	121 [54–899]	121 [54–975]	97,92% (47/48)
4	Performing a expert colposcopy (Guideline QI) (TV: > = 95%)	2149/ 2170	42 [16–205]	42 [16–211]	94,87% (37/39)	2056/ 2084	41 [5–234]	42 [9–234]	91,89% (34/37)	/	/		/
5	Abnormal findings Excision (TV: > = 85%)	1926/ 2081	36 [15–200]	41,5 [16–211]	81,58% (31/38)	1553/ 1724	36 [7–143]	41 [9–150]	74,29% (26/35)	/	/		/
6	Details pathology report (TV: > = 95%)	2291/ 2298	49 [16–211]	49 [16–211]	97,44% (38/39)	2025/ 2111	44 [0–234]	44 [14–234]	97,14% (34/35)	/	/		/
7	Proportion of R0 resection for CIN III (TV: > = 80%)	1345/ 1550	28 [9–135]	33 [10–150]	86,84% (33/48)	1176/ 1373	27.5 [4–124]	30.5 [6–150]	88,89% (32/36)	/	/		/
8	Follow-up care after excision (TV: > = 90%)	2129/ 2140	43,5 [15–211]	43,5 [16–211]	97,37% (37/38)	1885/ 1958	40,5 [8–234]	41,5 [9–234]	94,44% (34/36)	/	/		/
9	Proportion of knife conization to excisions (Guideline QI) (TV: < = 10%)	0/ 2170	0 [0–0]	42 [16–211]	100,00% (39/39)	6/ 2060	0 [0–3]	42,5 [9–234]	100,00% (36/36)	/	/		/

*TV *target value

#### QI 1: Presentation at tumour board

**Fig. 1:**
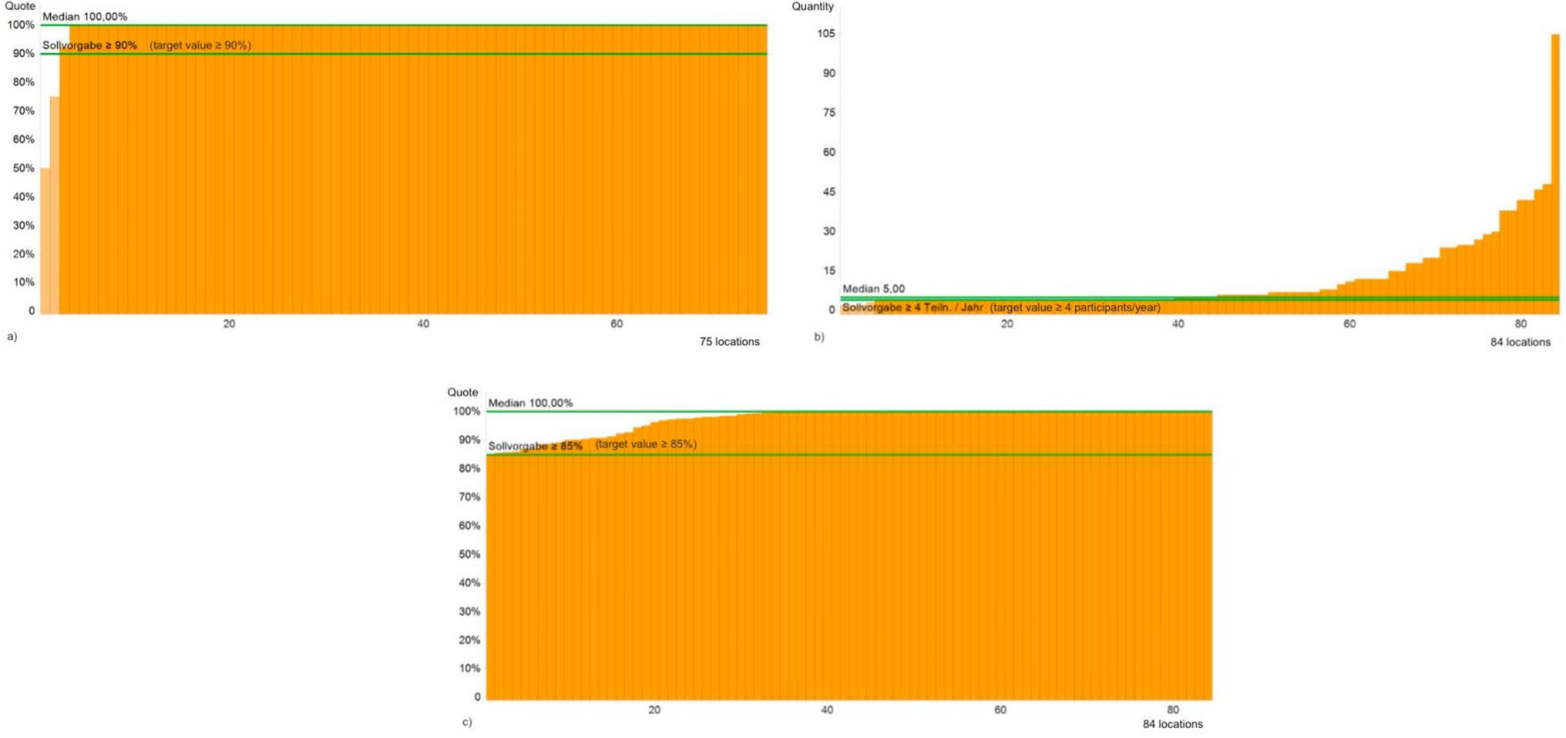
QIs 1 to 3—**a** Presentation at tumour board, **b** Participation in interdisciplinary tumour board/centre event, **c** Documentation (at least one sketch) of the visibility of the squamous cylindrical epithelial border

This QI covers all women with an invasive cancer who are presented to the interdisciplinary tumour board of a gynaecological cancer centre. The target of ≥ 90% was achieved with 73 dysplasia consultancies. 75 consultancies (89.29%) had evaluable data (Fig. 1a).

#### QI 2: Participation in interdisciplinary tumour board/ gynaecological cancer centre event

For the indicator of “participation in interdisciplinary tumour board /meeting of the centre”, the target was ≥ 4 participations per year. The median number of tumour boards or cancer centre events attended by the consultancies was five (range 2–105). The target was to attend at least four tumour boards or centre events. 80 consultancies (95.24%) met the target (Fig. 1b).

#### QI 3: Documentation (at least one sketch) of the visibility of the squamous cylindrical epithelial border

Since 2017, the proportion of documentation of visibility of the squamous cylindrical epithelial border has remained stable at a very high level (2017–2021: median 100%). In 2021, the target of ≥ 85% was achieved by all dysplasia consultancies (Fig. 1c).

QIs four to nine are reported voluntarily by the dysplasia consultancies. Therefore, the following QIs are not reported by all 84 consultancies.

#### QI 4: Performance of expert colposcopy

The performance indicator for expert colposcopy is a guideline-based quality indicator. Only 39 dysplasia consultancies (46.43% of 84 consultancies) provided evaluable data. 37 (94.87%) consultancies met the target of ≥ 95%.

#### QI 5: Abnormal findings (> = CIN 2) of excision

38 (45.24%) dysplasia consultancies provided data on the abnormal findings at excision metric. Of these, 31 (81.58%) were able to meet the target of ≥ 85%. The median remained largely stable from 92.31% in 2020 to 91.07% in 2021.

#### QI 6: Details pathology report

Data were submitted by 46.43% of dysplasia consultancies. 97.44% met the target of ≥ 95%. The proportion of women with a complete pathology report has remained stable at a very high level (median 100% in 2020 and 2021).

#### QI 7: Proportion of R0 resection for CIN III

Another indicator is the proportion of R0 resections in CIN III. 38 consultancies submitted their data and 33 (86,84%) were able to meet the target of ≥ 80%. The median remained stable at 86,50% in 2020 and 86,32% in 2021.

#### QI 8: Follow-up care after excision

Follow-up data for patients 6–12 months after excision were provided by 38 consultancies. 97.37% achieved the target of ≥ 90%. A median of 100% was achieved in 2020 and 2021.

#### QI 9: Proportion of cold knife conization of excisions

The guideline-based indicator "Proportion of cold knife conization in excisions" can also be reported voluntarily by dysplasia consultancies. Thirty-nine consultancies did so, and all met the target of ≤ 10%. No cold knife conisations were performed by dysplasia consultancies in any of the years (2017, 2020, 2021).

### Annual report of certified gynaecologic dysplasia units [15]

The annual report includes 40 of the 42 certified dysplasia units. Excluded are two dysplasia units that have suspended their certification in 2022 due to failure to meet qualitative and quantitative requirements. The QI data of the dysplasia units from 2017 to 2021 are shown in Table 3.

**Table 3:** Quality Indicators for dysplasia units (treatment years 2017–2021)

Quality indicator		2021				2020				2019				2018				2017			
		Results/cases	Median nominator	Median denominator	Centres meeting (TV)	Results/cases	Median nominator	Median denominator	Centres meeting (TV)	Results/cases	Median nominator	Median denominator	Centres meeting (TV)	Results/cases	Median nominator	Median denominator	Centres meeting (TV)	Results/cases	Median nominator	Median denominator	Centres meeting (TV)
1	Tumour board presentation (TV: > = 90%)	877/ 889	15,5 [5–85]	15,5 [5–85]	100,00% (40/40)	1363/ 1374	19 [5–254]	19 [5–254]	97,44% (38/39)	1507/ 1514	32 [2–271]	32 [2–271]	100,00% (37/37)	1572/ 1582	23 [5–254]	23 [5–254]	100,00% (35/35)	1513/ 1534	27 [5–228]	27 [5–228]	96,67% (29/30)
2	Participation in interdisciplinary tumour board/ cancer centre event (TV: > = 4 participants/year)	1147	28 [0–112]		97,50% (39/40)	1021	23 [8–91]		100,00% (39/39)	1131	25 [6–135]		97,30% (36/37)	988	25 [8–120]		100,00% (35/35)	667	19.5 [8–51]		100,00% (30/30)
3	Documentation (at least one sketch) of the visibility of the squamous cylindrical epithelial border (TV: > = 85%)	39,627/ 40,635	794 [272–3105]	810,5 [272–3120]	100,00% (40/40)	29,494/ 30,425	614 [252–2600]	614 [270–2630]	100,00% (39/39)	26,514/ 27,168	546 [187–2160]	557 [192–2180]	100,00% (37/37)	21,867/ 22,550	491 [185–2130]	505 [187–2163]	100,00% (35/35)	17,526/ 17,904	467 [153–1375]	481 [153–1412]	100,00% (30/30)
4	Performing a expert colposcopy (Guideline QI) (TV: > = 95%)	8812/ 8859	170,5 [61–657]	178 [62–657]	100,00% (40/40)	8021/ 8106	174 [58–677]	176 [58–677]	94,87% (37/39)	6428/ 6497	128 [52–718]	129 [54–718]	100,00% (37/37)	5766/ 5806	145 [64–562]	149 [66–562]	100,00% (35/35)	5289/ 5350	154,5 [67–460]	157 [67–460]	100,00% (30/30)
5	Abnormal findings Excision (TV: > = 85%)	7865/ 8859	150 [61–612]	178 [62–657]	80,00% (32/40)	7145/ 8106	149 [51–625]	176 [58–677]	79,49% (31/39)	5786/ 6497	113 [49–661]	129 [54–718]	78,38% (29/37)	5041/ 5806	120 [61–484]	149 [66–562]	71,43% (25/35)	4716/ 5350	149,5 [62–433]	157 [67–460]	80,00% (24/30)
6	Details pathology report (TV: > = 95%)	10,075/ 10,137	213,5 [80–688]	213,5 [80–697]	97,50% (39/40)	9538/ 9636	214 [60–855]	214 [60–859]	97,44% (38/39)	8206/ 8252	178 [79–896]	178 [79–908]	97,30% (36/37)	8189/ 8225	194 [88–911]	194 [92–923]	100,00% (35/35)	6805/ 6821	187.5 [99–521]	187.5 [100–521]	100,00% (30/30)
7	Proportion of R0 resection for CIN III (TV: > = 80%)	4786/ 5614	91 [21–349]	118 [37–397]	87,50% (35/40)	4292/ 5029	103 [31–241]	117 [36–287]	84,62% (33/39)	3640/ 4222	79 [23–364]	97 [28–410]	83,78% (31/37)	3409/ 3928	79 [20–310]	93 [24–356]	91,43% (32/35)	3134/ 3590	87 [39–292]	96 [48–302]	90,00% (27/30)
8	Follow-up care after excision (TV: > = 90%)	8773/ 8859	175 [56–657]	178 [62–657]	100,00% (40/40)	8027/ 8106	176 [58–677]	176 [58–677]	97,44% (38/39)	6448/ 6497	129 [53–718]	129 [54–718]	100,00% (37/37)	5786/ 5806	145 [59–562]	149 [66–562]	97,14% (34/35)	5282/ 5350	156 [67–460]	157 [67–460]	96,67% (29/30)
9	Proportion of knife conization to excisions (Guideline QI) (TV: < = 10%)	3/ 8106	0 [0–3]	178 [62–657]	100,00% (40/40)	3/ 8859	0 [0–3]	176 [58–677]	100,00% (39/39)	4/ 6497	0 [0–4]	129 [54–718]	100,00% (37/37)	6/ 5806	0 [0–4]	149 [66–562]	100,00% (35/35)	6/ 5350	0 [0–3]	157 [67–460]	100,00% (30/30)

*TV *target value

#### QI 1: Presentation at tumour board

Since 2017, the proportion of cases of invasive cervical cancer presented to the tumour board of the gynaecologoical cancer centre has remained stable at a very high level (2017–2020: median 100%). In 2020, all units reached the target of 90% or more. All dysplasia units met the target of ≥ 90%.

#### QI 2: Participation in interdisciplinary tumour board

**Fig. 2:**
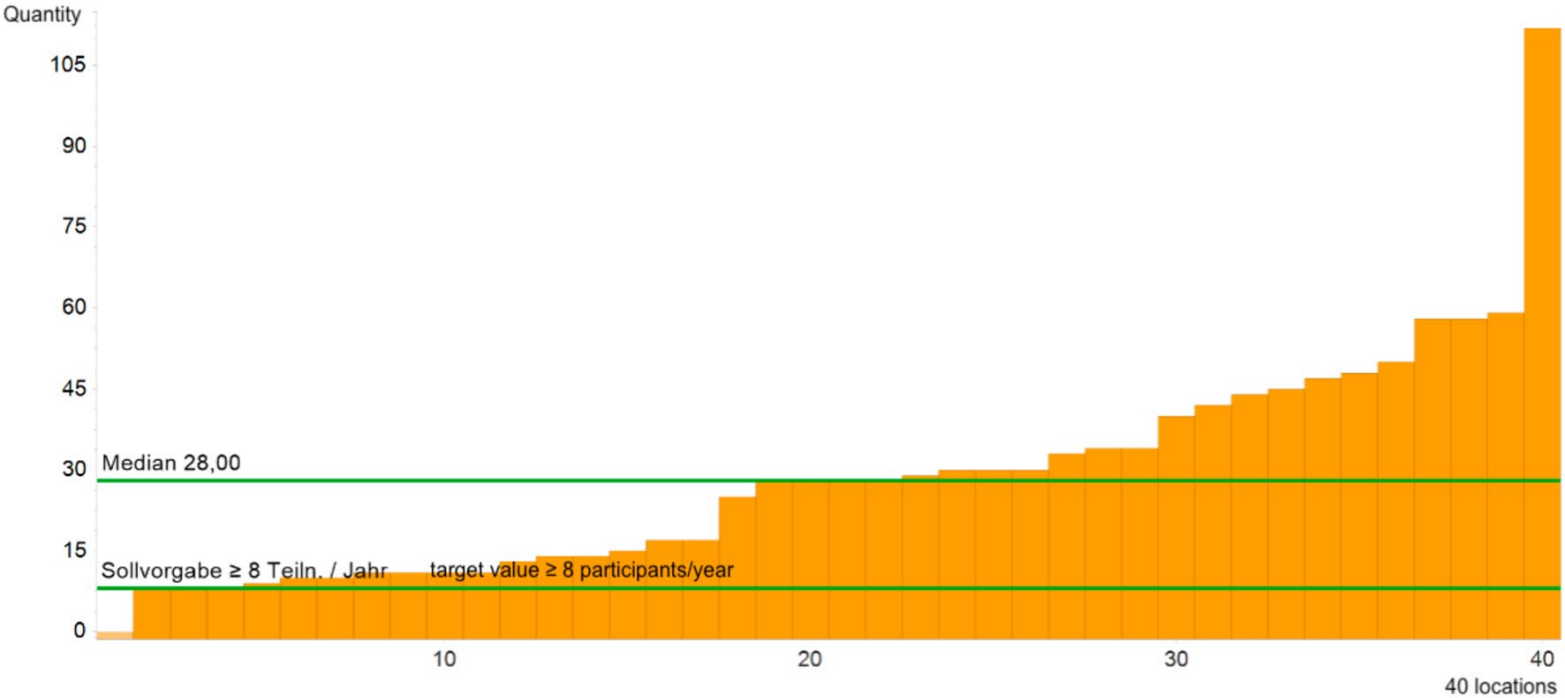
QI of participation in interdisciplinary tumour board

The target of ≥ 8 participations in interdisciplinary tumour board per year was achieved by 97.50% of the dysplasia units. All units provided data. After a slight decrease in the first Covid year, the frequency of participation in the tumour board of the Gynaecological Cancer Centre increased again in 2021 (Fig. 2).

#### QI3: Documentation (at least one sketch) of the visibility of the squamous cylindrical epithelial border

The target of ≥ 85% was met by all 40 units. The median and minimal values remained stable at a high level (99,08% in 2017 to 98,62% in 2021 and 88,84% in 2017 to 88,70% in 2021, respectively).

#### QI 4: Performance of expert colposcopy

All units provided data on the guideline-based metric for performing expert colposcopy. The target of ≥ 95% was achieved by all dysplasia units. Expert colposcopy was performed preoperatively in almost all patients undergoing excision (8812 of 8859 patients). The median was stable over the years (100% in 2017 to 2021).

#### QI 5: Abnormal findings of excision

**Fig. 3:**
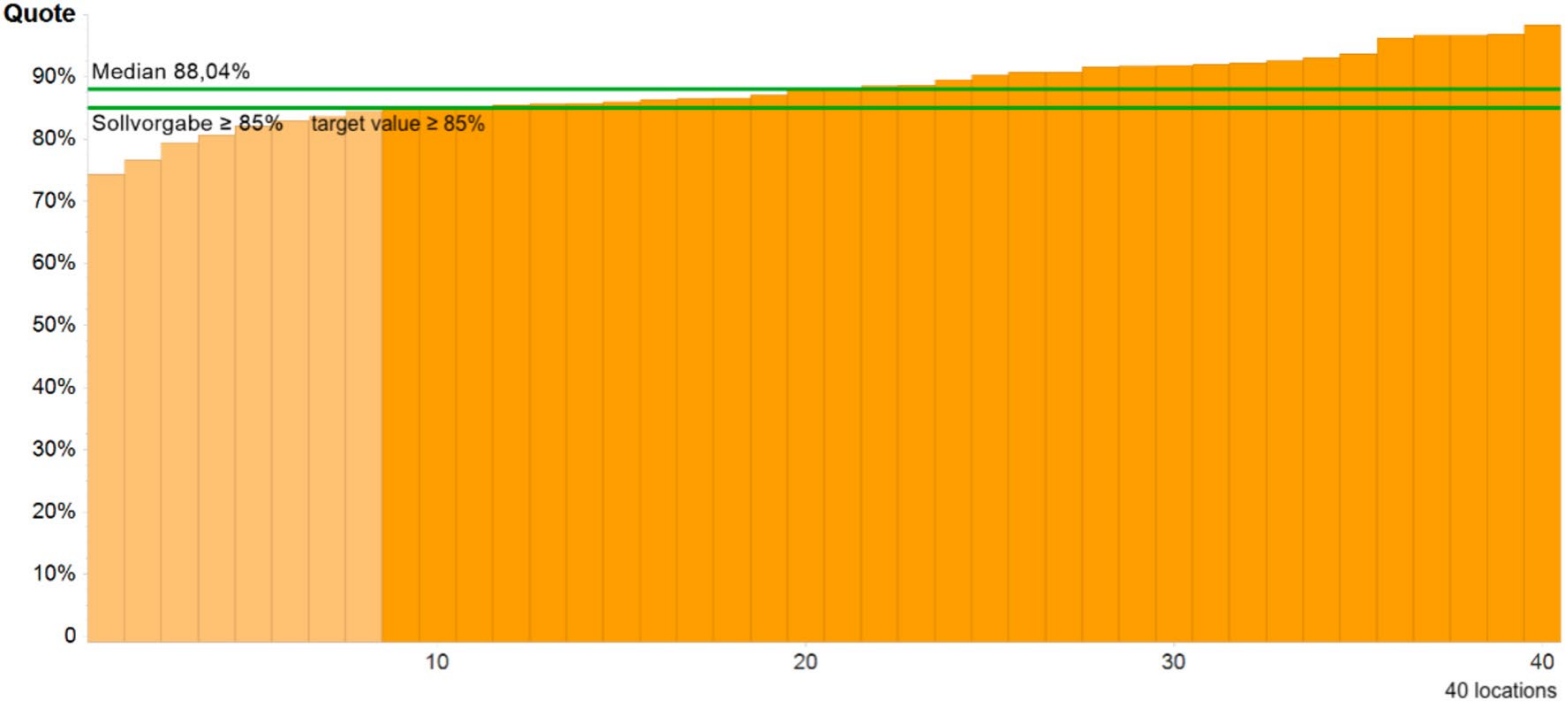
QI of abnormal findings Excision

Excision of abnormal findings equal to or greater than CIN 2 is a mandatory indicator for dysplasia units compared to dysplasia consultancies. All 40 dysplasia units provided their data but only 80% of them were able to meet the target of ≥ 85%. In 8 units, the target of at least 85% >  = CIN II lesions after excision was just missed (Fig. 3).

#### QI 6: Details pathology report

**Fig. 4:**
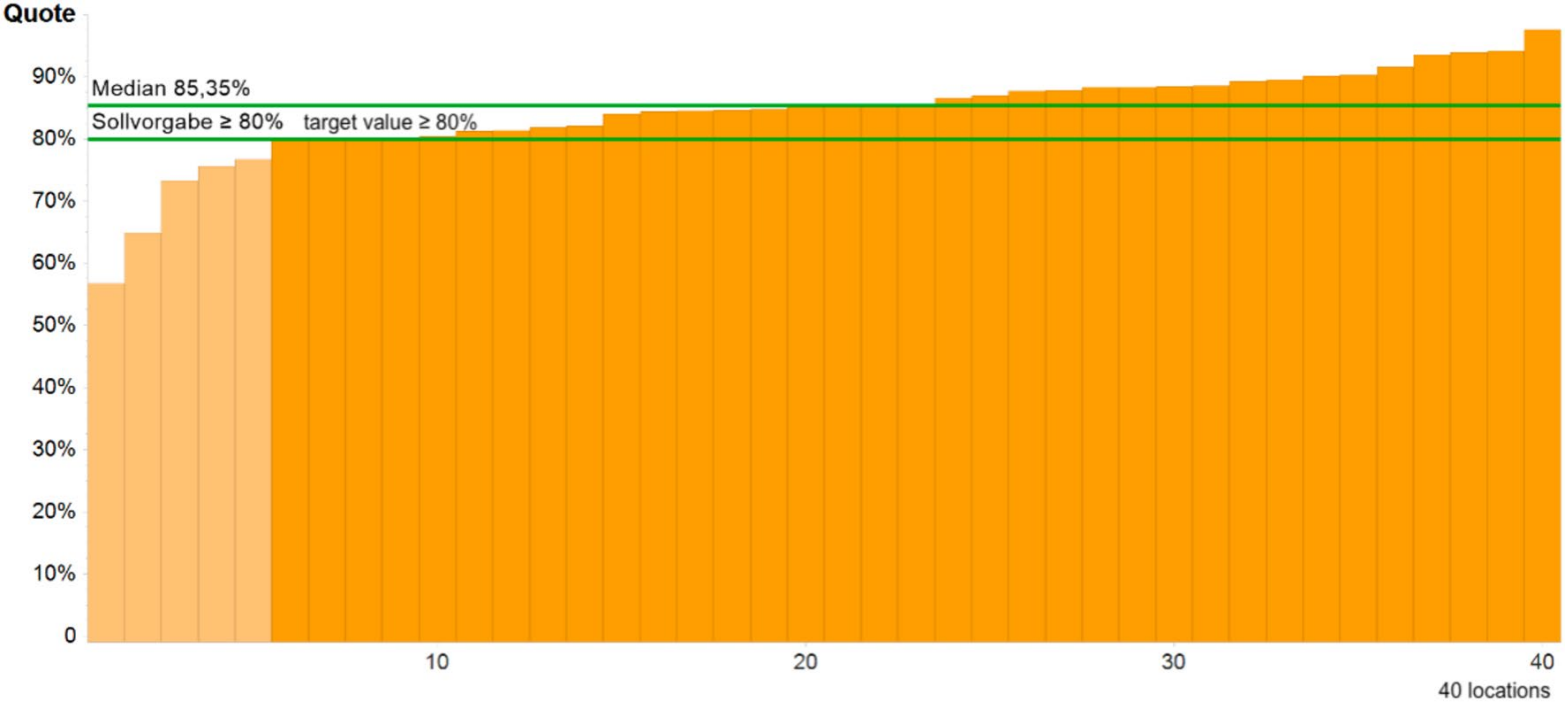
QI of the proportion of R0 resection for CIN III

The sixth metric, completeness of the report, was met in all dysplasia units. 39 out of 40 units achieved the target ≥ 95%. Complete findings reports were found in almost all patients with excision (1,0075 of 1,0137 patients) (Fig. 4).

#### QI 7: Proportion of R0 resection for CIN III

The target of ≥ 80% for the ratio of R0 resection rate for CIN III was achieved by 87.50% of dysplasia units. 5 dysplasia units did not meet the target of at least 80% R0 resection for CIN III.

#### QI 8: Follow-up care after excision

All dysplasia units met the target for the metric of follow-up after excision. 8,773 out of 8,859 patients with excision received a recommendation for a single follow-up at 6–12 months. The median remained at a high level of 100% from the year 2017 to 2021.

#### QI 9: Proportion of cold knife conization of excisions

There is a target of ≤ 10% for the ratio of knife conization to excisions. All units met this target.

Only 3 of the 8859 patients who underwent knife conization. All dysplasia units therefore remain below the target of 10%.

### Comparison of dysplasia consultation and unit data

As dysplasia consultancies are only audited every three years, comparisons between years are difficult. Data could be compared every three years. However, it is important to note that not all consultancies are recertified and that new consultancies are certified. Furthermore, only the first three indicators can be used for comparison, as dysplasia consultancies are not required to report indicators four to nine. The data are shown in Table 2.

The annual audit makes it possible to compare data from dysplasia units over the years. The number of certified dysplasia units increased steadily from 2017 to 2021 from 30 to 40. Likewise, the number of patients with dysplasia treated in dysplasia units increased from 5806 to 8859 (Table 2). Not all units could be certified in 2019 and 2021. Excluded are dysplasia units that had their certification suspended in those years because they did not meet the qualitative and quantitative requirements.

Over the years, the fulfilment of QI targets have remained largely constant. We have seen an increase in the target achievement for the first indicator, tumour board presentation and the eighth indicator of follow-up after excision. In contrast, there has been a deterioration in the performance of indicators two, six and seven (participation in interdisciplinary tumour board, information on report of findings, proportion of R0 resection in CIN III).

## Discussion

To the best of our knowledge, this is the first paper to provide an overview of the implementation status and development of QI in certified dysplasia units and consultancies. This paper provides a differentiated overview of the statistics of the QIs of the dysplasia units and consultancies. QIs are an important tool for quality management. The aim of their use is the continuous improvement of care. The presented selection of QIs was developed according to the methodology of the German Guideline Program in Oncology (GGPO) and the Certification Commission of the German Cancer Society [16]. Our findings demonstrate that the evaluated QIs are implemented to a very high degree. The quality of care is highlighted. The results of the dysplasia units and consultancies are presented over time (2017 to 2021). This analysis shows the quality of care and thus the whole cervical cancer screening program. By certifying and reviewing the metrics of gynaecological dysplasia consultancies and units, the cohesiveness of health care from prevention and early diagnosis to treatment in gynaecological cancers centres could be assured for the first time. The monitoring of quality indicators ensures an expertise in cervical screening in the units and consultancies. Since the introduction of the QIs were introduced as part of the certification of units and consultancies, they have been successfully met and as our data show, maintained over time. This is guaranteed, for example, by standard operating procedures and instructions. If an unjustifiable non-fulfilment of a QI is repeated, a “deviation” will be assigned as part of the audit. If the QI is ultimately not met, a certificate cannot be issued, or the dysplasia unit or consultancy can be withdrawn. This has happened twice. This obviously excludes the non-mandatory QIs 4–9 for the dysplasia consultancies. Nevertheless, when evaluating and auditing QIs, care should be taken to ensure that failure to meet the target value of a QI does not necessarily indicate inadequate provider performance. It should be noted that situations in routine care can be very complex, making it impossible to draw conclusions from QIs without further background information [17, 18]. The frequency of dysplasia unit attendance at the Gynaecologic Cancer Centre tumour board (QI 2) decreased slightly in the first year of Covid. In 2021, however, attendance is increasing again. The trend in dysplasia unit attendance at tumour board continues to increase, suggesting improved collaboration that is integrated into routine practice. One unit did not participate in the tumour board, citing a Covid-related ban on participation in external tumour board. At the same time, it is described that telephone arrangements were made with the management of the Gynaecologic Cancer Centre. Regarding QI 5 “Abnormal findings excision”, the target of at least 85% >  = CIN II lesions after excision of eight dysplasia units was missed by eight units. The auditors discussed the cases in detail with the units. Two main explanations are given as justifications. Firstly, conspicuous findings had already been completely removed by biopsy, and secondly, patients with CIN I who had completed family planning and had a higher need for safety, or their referring physician, wanted an excision. In addition, the units reported that CIN II + cases were found at follow-up examinations by the pathology department, so that an SOP had been developed for mandatory follow-up in the case of negative postoperative histology. Five dysplasia units did not meet the target of at least 80% R0 resections for CIN III (QI 7). The auditors or the “Certificate Award” Committee issue deviations or reduce the duration of the certificate. The cases are discussed intensively in the sense of an individual case analysis and with the determination of measures on-site. Considering the need for gentle resection in young patients, the following measures have been agreed upon. These include destructive post-treatment of the ectocervical margins using colposcope during surgery, training of the surgeons, definition of smaller surgical teams, more generous excision in patients with completed family planning. However, the results of QI in the certification process can be used to identify areas for improvement. Dysplasia units or consultancies that do not meet the targets have the opportunity to justify and discuss the deviation during the audits. This ensures that adequate actions are agreed upon between the units and the auditors to improve QI results [19, 20]. In the following year, these discussed measures can be reviewed as part of the audit. This ensures an effective quality improvement process based on the QI guideline in the certified units. Systematic implementation and evaluation of QI can help to increase knowledge by generating data to improve the care and prevention of affected women [20].

## Conclusion

The incidence of cervical cancer has fallen significantly in recent decades. This is mainly due to improved screening. The targets for the four QIs were achieved by both the dysplasia consultancies and the units in at least 95% of the certified centres (QI 1: 100%, QI 2: 95%, QI 3: 100%; QI 1: 100%, QI 2: 97%, QI 3: 100%, respectively). With the certification of gynaecological dysplasia consultancies /units, the continuum of healthcare from prevention to treatment of gynaecological cancers in certified gynaecological cancer centres was ensured for the first time. The monitoring of defined quality indicators is intended to ensure that the quality of care in the dysplasia consultancies and units is guaranteed. QIs can be used to establish guideline-compliant treatment and motivate practitioners to review their treatment outcomes. The results of the QI reports are reported to the medical guideline development groups and provide information on whether and how the recommendations made are implemented in everyday clinical practice. They thus provide a stimulus for the further development of the corresponding guideline.

## References

[1] Bedell SL, Goldstein LS, Goldstein AR, Goldstein AT (2020) Cervical cancer screening: past, present, and future. Sex Med Rev 8(1):28–37 31791846 10.1016/j.sxmr.2019.09.005

[2] Koch-Institut R (2019) Gebärmutterhalskrebs (Zervixkarzinom), updated 30.09.2022. Available from: https://www.krebsdaten.de/Krebs/DE/Content/Krebsarten/Gebaermutterhalskrebs/gebaermutterhalskrebs_node.html.Accessed 04 Aug 2024

[3] Stuebs FA, Koch MC, Dietl AK, Adler W, Geppert C, Hartmann A et al (2022) Cytology and high-risk human papillomavirus test for cervical cancer screening assessment. Diagnostics (Basel) 12(7):1748 35885651 10.3390/diagnostics12071748 PMC9318141

[4] Bujan Rivera J, Klug SJ (2018) Cervical cancer screening in Germany. Bundesgesundheitsblatt Gesundheitsforschung Gesundheitsschutz 61(12):1528–1535 30397722 10.1007/s00103-018-2835-7

[5] Beckmann MW, Stubs FA, Koch MC, Mallmann P, Dannecker C, Dietl A et al (2022) Diagnosis, Therapy and follow-up of cervical cancer guideline of the DGGG, DKG and DKH S3-Level, AWMF epidemiology screening diagnostics and therapy. Geburtshilfe Frauenheilkd 82(2):139–180 35169387 10.1055/a-1671-2158 PMC8837407

[6] Fehm T, Stubs FA, Koch MC, Mallmann P, Dannecker C, Dietl A et al (2022) Diagnosis, therapy and follow-up of cervical cancer. Guideline of the DGGG, DKG and DKH (S3-Level, AWMF registry no. 032/033OL, May 2021)-part 2 with recommendations on psycho-oncology, rehabilitation, follow-up, recurrence, palliative therapy and healthcare facilities. Geburtshilfe Frauenheilkd 82(2):181–205 35197803 10.1055/a-1671-2446 PMC8855983

[7] Bundesausschuss G (2018) Richtlinie für organisierte Krebsfrüherkennungsprogramme und Krebsfrüherkennungs-Richtlinie: Programm zur Früherkennung von Zervixkarzinomen

[8] Langer T, Follmann M (2015) The German guideline program in oncology (GGPO): a central core of an evidence-based, patient-centered interdisciplinary oncology? Z Evid Fortbild Qual Gesundhwes 109(6):437–444 26474648 10.1016/j.zefq.2015.09.007

[9] Thomas Langer SW, Christoph Kowalski (2017) Qualitätsindikatoren in der Onkologie. Versorgungsforschung

[10] Beckmann MW, Quaas J, Bischofberger A, Kammerle A, Lux MP, Wesselmann S (2014) Establishment of the certification system “gynaecological dysplasia” in Germany. Geburtshilfe Frauenheilkd 74(9):860–867 25278628 10.1055/s-0034-1383042 PMC4175128

[11] Colposcopy EFF. The EFC Quality and Standards Group 2020. Available from: https://efcolposcopy.eu/quality-and-standards-group/. Accessed 04 Aug 2024

[12] Candice A, Tedeschi N (2013) The requirements in this booklet are effective for any mentee applying for the colposcopy mentorship program on or after March 21. Available from: https://www.asccp.org/Assets/7494717c-98c0-4d70-81cc-0aafebf017ed/635947807358470000/asccp-cmp-booklet-2013-pdf. Accessed 04 Aug 2024

[13] J. Quaas AB, M.P. Lux, S. Wesselmann, M.W. Beckmann (2015) Zertifizierungsprogramm für Gynäkologische Dysplasie-Sprechstunden. DKG-aktuell. 1–4.

[14] e.V. DK, e.V. DGfGuG, e.V. AGO, e.V. AZuK, Krebszentren ZG (2023) Jahresbericht der zertifizierten Gynäkologischen Dysplasie-Sprechstunden - Kennzahlenauswertung. Available from: https://www.krebsgesellschaft.de/jahresberichte.html?file=files/dkg/deutsche-krebsgesellschaft/content/pdf/Zertifizierung/Jahresberichte%20mit%20DOI%20und%20ISBN/qualitaetsindikatoren_gynaekologische-dysplasien_2023-A1_230526.pdf&cid=112679. Accessed 04 Aug 2024]

[15] e.V. DK, e.V. DGfGuG, e.V. AGO, e.V. AZuK, Krebszentren ZG (2023) Jahresbericht der zertifizierten Gynäkologischen Dysplasie-Einheiten - Kennzahlenauswertung. Available from: https://www.krebsgesellschaft.de/jahresberichte.html?file=files/dkg/deutsche-krebsgesellschaft/content/pdf/Zertifizierung/Jahresberichte%20mit%20DOI%20und%20ISBN/qualitaetsindikatoren_gynaekologische-dysplasien_2023-A1_230526.pdf&cid=112679. Accessed 04 Aug 2024

[16] Leitlinienprogramm Onkologie (Deutsche Krebsgesellschaft DK, AWMF): S3-Leitlinie Prävention des Zervixkarzinoms, Langversion 1.1, 2020, AWMF Registernummer: 015/027OL 2020 [Available from: http://www.leitlinienprogramm-onkologie.de/leitlinien/zervixkarzinom-praevention/

[17] Griesshammer E, Wesselmann S, Beckmann MW, Dannecker C, Wagner U, Sibert NT et al (2023) Quality assurance and improvement in oncology using guideline-derived quality indicators - results of gynaecological cancer centres certified by the German cancer society (DKG). J Cancer Res Clin Oncol 149(5):1703–1715 35657567 10.1007/s00432-022-04060-8 PMC10097788

[18] Junor EJ, Hole DJ, McNulty L, Mason M, Young J (1999) Specialist gynaecologists and survival outcome in ovarian cancer: a Scottish national study of 1866 patients. Br J Obstet Gynaecol 106(11):1130–1136 10549956 10.1111/j.1471-0528.1999.tb08137.x

[19] Ruckher J, Lobitz J, Follmann M, Derenz S, Schmidt S, Mensah J et al (2022) Guideline-based quality indicators for kidney and bladder cancer in Germany: development and implementation. Urol Int 106(4):360–367 34384078 10.1159/000517893

[20] Stuebs FA, Beckmann MW, Fehm T, Dannecker C, Follmann M, Langer T et al (2023) Implementation and update of guideline-derived quality indicators for cervical cancer in gynecological cancer centers certified by the German cancer society (DKG). J Cancer Res Clin Oncol. 10.1007/s00432-024-05769-4 37452203 10.1007/s00432-024-05769-4 PMC10587177

